# Stochastic Geometric-Based Modeling for Partial Offloading Task Computing in Edge-AI Systems

**DOI:** 10.3390/s25226892

**Published:** 2025-11-12

**Authors:** Hamid Saeedi, Ali Nouruzi

**Affiliations:** College of Engineering and Technology, University of Doha for Science and Technology, Doha P.O. Box 24449, Qatar; ali.nouruzi@udst.edu.qa

**Keywords:** resource allocation, multi-access edge computing, edge AI, partial offloading

## Abstract

This paper proposes a cooperative framework for resource allocation in multi-access edge computing (MEC) under a partial task offloading setting, addressing the joint challenges of learning performance and system efficiency in heterogeneous edge environments. In the proposed architecture, selected users act as edge servers (SEs) that collaboratively assist others alongside a central server (CS). A joint optimization problem is formulated to integrate model training with resource allocation while accounting for data freshness and spatial correlation among user tasks. The correlation-aware formulation penalizes outdated and redundant data, leading to improved robustness against non-i.i.d. distributions. To solve the NP-hard problem efficiently, a projected gradient descent (PGD) method is developed. The simulation results demonstrate that the proposed cooperative approach achieves a balanced delay of 0.042 s, close to edge-only computing (0.033 s) and 30% lower than the CS-only mode, while improving clustering accuracy to 99.2% (up to 15% higher than the baseline). Moreover, it reduces the central server load by nearly half, ensuring scalability and latency compliance within 3GPP limits. These findings confirm that cooperation between SEs and the CS substantially enhances reliability and performance in distributed Edge-AI system.

## 1. Introduction

### 1.1. State-of-the-Art and Motivations

Recent advances in artificial intelligence (AI) have sparked innovation in various technological domains. Most conventional AI solutions rely on centralized computing architectures that depend on large-scale data collection. While these centralized models offer powerful learning, perception, and decision-making capabilities, they have their own limitations in dynamic and uncertain environments. Transferring data to a central server and accumulating it for computing is time-consuming and costly. Additionally, providing adequate computing resources on a central server is challenging, especially under high demand or time-sensitive conditions. To address these challenges, researchers have proposed more distributed and adaptive architectures. One prominent approach is multi-access edge computing (MEC), which brings computing and storage resources closer to end devices at the edge of the network. This configuration improves task offloading efficiency and enables real-time system responsiveness. Edge AI enables the execution of AI models directly at the network edge, allowing real-time decision-making with reduced latency and lower reliance on centralized servers. This approach is particularly valuable in time-sensitive and resource-constrained environments [1,2]. Due to the significant advantages of Edge AI, such as enhanced operational efficiency, improved data privacy, and ultra-low latency, global interest and investment in this field have surged in recent years. According to market analysis, the global Edge AI market is projected to exceed USD 66 billion by 2030 [3,4,5].

Despite its benefits, Edge AI and MEC are faced with several significant challenges. One of the most critical issues is the propagation of uncertainty in distributed environments. In this regard, stochastic geometry models can provide a powerful analytical framework for characterizing spatial randomness and evaluating network performance [6,7,8,9,10]. Furthermore, due to asynchronous updates and decentralized model execution, local errors can accumulate and spread across the network, ultimately degrading the overall model accuracy and reliability. Another major challenge lies in the non-independent and identically distributed (non-i.i.d) nature of user data. While various solutions have been proposed in the context of federated learning (FL) to mitigate this issue, concerns remain regarding the generalization and reliability of global models trained on highly heterogeneous local datasets [11]. Such data distribution disparities can significantly disrupt model convergence and performance. While most existing works on non-i.i.d data in FL and machine learning (ML) focus on non-uniformity of local data distributions (e.g., label skew or quantity skew), fewer studies address the non-independence aspect, where user data may be statistically correlated or dependent across clients [12,13,14].

These challenges highlight the need for robust and correlation-aware learning frameworks that maintain accuracy, adaptability, and convergence under decentralized and statistically dependent conditions. In such environments, traditional centralized or federated approaches often fall short, particularly when dealing with heterogeneous and temporally dynamic data [15,16,17].

To address these limitations, we propose a distributed learning framework tailored for partial task offloading in MEC environments. Unlike conventional paradigms, our approach enables user devices to selectively offload task-related data to edge or central servers (CS) via data-level cooperation. This setup leverages local computation, respects delay constraints, and accommodates statistical heterogeneity across users. A key novelty of our model lies in its integration of spatial and directional task correlations. This is often overlooked in existing works, i.e, the data is treated as being i.i.d which is not an accurate assumption. We introduce a correlation-aware loss function that explicitly incorporates data freshness and penalizes contributions from distant or weakly correlated users. This design enhances the relevance of updates, reduces the impact of outdated or biased data, and improves overall learning robustness in Edge AI systems. Using stochastic geometry analysis, we can analytically guarantee that users can be served by at least one local server at any time. In summary, the main question addressed in this paper is as follows: *How can user tasks in a network be served efficiently under a partial offloading MEC scenario, with priority for edge servers, while taking into account the characteristics of an AI-driven edge model, including communication latency and the challenges posed by non-i.i.d data?*

### 1.2. Contributions

Different from previous works [18,19,20,21,22,23], this paper introduces a novel cooperative computation framework for partial offloading in MEC environments. The main contributions are as follows:

- We propose a cooperative partial offloading model in MEC environments, where tasks can be processed either locally by neighboring users or centrally by a server, taking spatial and directional correlations into account where a closed-form upper bound for spatial correlation is derived to constrain offloading decisions based on user proximity and sensing overlap.
- To minimize learning loss under delay and resource constraints, a novel optimization problem is formulated that incorporates freshness-aware weighting, correlation modeling, and allocation decisions. Moreover, to improve robustness in non-i.i.d. settings, we integrate earth mover’s distance (EMD) into the loss function to capture distributional dissimilarity among users’ data.
- To ensure scalability, we develop a coordination-free solution method suitable for practical deployment in distributed MEC systems.
- Leveraging stochastic geometry, we provide tractable analytical characterizations of coverage probability and delay distribution, which not only enable probabilistic guarantees on task offloading but also ensure the generalizability of the proposed framework to large-scale and heterogeneous MEC networks.
- We show that using the proposed framework, we can significantly reduce the computation load on the central server compared to baseline schemes and for a given delay threshold, we can give service to considerably higher number of users.

## 2. Related Works

In this section, we first review related works on MEC systems and their resource management strategies, followed by a discussion on studies focusing on Edge AI. Regardless of the method used to solve the optimization problem in MEC-based systems, task offloading is typically classified as either full or partial. In full offloading, the entire task is computed at the edge server, while in partial offloading, the task is split between local and edge computing server to balance latency and resource usage [24,25,26]. In [27] the authors investigate parallel task offloading in fog-enabled IoT networks, where computational tasks are divided into multiple sub-tasks and executed concurrently across heterogeneous fog nodes. They formulate the resource allocation problem with the objective of minimizing overall task latency while ensuring simultaneous completion of all sub-tasks, which is essential for efficient utilization of distributed resources. To address the inherent instability caused by interdependent task assignments, the authors design a matching-based allocation framework that maintains stable associations between task-originating devices and helper nodes. Through extensive simulations, the proposed method is shown to reduce average latency by up to 52% under high workload conditions, outperforming several state-of-the-art offloading strategies and demonstrating its suitability for large-scale, delay-sensitive IoT systems. In [20] the authors propose a computation offloading scheme for IoT applications involving dependent tasks. This scheme consists of two main components: a multi-queue priority algorithm that schedules dependent sub-tasks, and a deep reinforcement learning method based on Actor–Critic for making dynamic offloading decisions. The framework is designed to minimize task completion time and energy consumption in edge environments by handling task dependencies and fluctuating network conditions efficiently. By leveraging the Lyapunov method, the stochastic optimization problem is stated in  [22] with the aim of providing a joint task offloading and resource allocation framework for edge-assisted machine learning inference. This framework focuses on minimizing end-to-end latency while preserving inference accuracy and queue stability. The model considers local and edge inference options, whereby each console can adapt the quality of uploaded data and dynamically decide on offloading and computing strategies. The problem is decomposed into three sub-problems: offloading and channel allocation; data quality adjustment; and computational resource assignment. These sub-problems are solved using convex optimization and low-complexity heuristic methods. The simulation results demonstrate substantial improvements in latency reduction and stability under dynamic edge environments.

In [28], the authors proposed a method called the Decentralized Distributed Sequential Neural Network (DDSNN), tailored for low-power devices in wireless sensor networks, where conventional deep models face strict memory and energy constraints. By sequentially partitioning a LeNet model across multiple nodes, DDSNN enables fully decentralized inference without the need for compression or centralized coordination. In a predictive maintenance case study involving industrial pump vibration data, the framework preserved full precision, achieved 99% accuracy, and reduced inference latency by nearly 50% compared to the baseline. Although the accuracy gain over the non-distributed model was marginal, the authors emphasize that in highly resource-constrained settings, even slight improvements are significant, making DDSNN a practical and scalable solution.

The approach proposed in [21] addresses FL in heterogeneous edge environments by allowing each edge device to perform a different number of local updates, adapting to their computational capacity. This idea lays a foundation for handling heterogeneity in edge systems and can be extended further to include other real-world factors. For instance, while the original model focuses on computational diversity, it does not account for communication delays or queuing effects that are common in distributed systems. Additionally, the influence of data heterogeneity is not explicitly modeled.

Authors in [18] introduce a dynamic client selection strategy for FL, aiming to improve training efficiency under resource constraints in edge environments. The approach incorporates client-side characteristics such as computation power and data volume into a selection metric to adaptively involve suitable participants in each communication round. This method improves convergence speed and reduces communication cost. However, the model assumes relatively consistent client availability and does not explicitly address delay variability or the impact of severe data heterogeneity, which are common in real-world edge networks. Moreover, the paper focuses primarily on client selection policies rather than modeling deeper aspects such as uncertainty propagation or the effects of delayed or imbalanced updates on global performance. These aspects open opportunities for extending this methodology toward more delay-aware and distribution-sensitive learning frameworks.

To improve personalization in FL, the authors in  [29] propose an adversarial training framework combined with data-free knowledge distillation. Their method leverages earth mover’s distance (EMD) to align local and global data distributions, effectively addressing the challenge of non-i.i.d data across clients while preserving privacy. Although this framework improves global model adaptation, it does not consider delay sensitivity, dynamic task partitioning, or computing constraints common in edge environments. Nevertheless, the idea presents a promising direction that can be extended to such realistic, delay-aware, and resource-constrained edge AI settings.

With the aim of addressing label distribution skew in FL, the authors in [30] propose a novel learning approach that leverages knowledge distillation and a label-invariant teacher-student framework. Their method focuses on mitigating performance degradation due to non-i.i.d label distributions without directly sharing model parameters. This work sheds light on the importance of decoupling label heterogeneity from the model optimization process. While the approach effectively addresses label skew, it can be extended to incorporate delay sensitivity and partial offloading in edge computing environments—dimensions must be considered.

Furthermore, authors in [31] have explored pruning-aware collaborative inference of large AI models at the edge, where model partitioning and resource optimization are jointly considered to balance accuracy, latency, and energy consumption. While this provides important insights into enabling efficient edge inference, it does not address aspects such as training under heterogeneous data distributions and stochastic task offloading, which remain central to advancing edge intelligence.

To facilitate a more effective comparison between this work and prior studies, we present Table 1.

***Symbol Notation:*** $f_{X}\left(x\right)$ and $F_{X}\left(x\right)$ denote the probability density function (PDF) and cumulative distribution function (CDF) of *x*. Pr(x) denotes the probability of *x*. |.| is the absolute value, and ⌈.⌉ is the ceiling function.

## 3. System Model and Parameters

**Figure 1:**
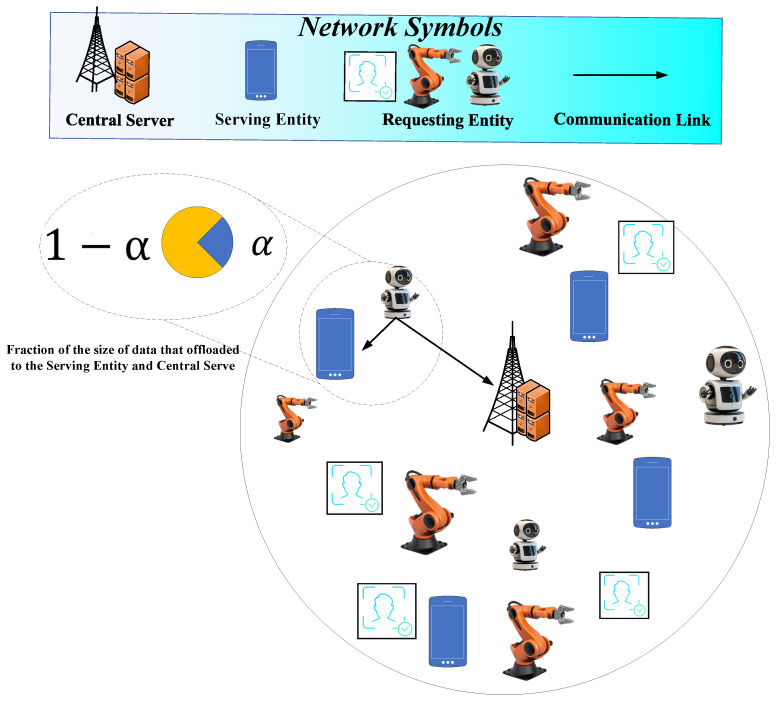
A hybrid edge architecture in which some entities request task execution (REs) and others provide computing resources (SEs). A CS is also available for additional offloading. Typical use cases include smart campuses or factories, where IoT devices and robots can be considered as REs, and where attendees smartphones can be considered as SEs performing partial task offloading.

This article focuses on a task offloading scenario in which data-generating equipment, referred to as requesting entities (REs), can delegate their computational tasks either to nearby local edge devices, referred to as serving entities (SEs), or to a centralized server (CS). The goal is to enable efficient and delay-sensitive learning by leveraging Edge AI frameworks, where training occurs across distributed nodes with limited resources and potentially heterogeneous data distributions. In this regard, as can be seen in Figure 1, we consider a central server for task computing.

The set of equipment whose tasks needed to be addressed is represented by $\mathrm{RE}={\left\{r\right\}}_{1}^{R}$. In addition, the set of equipment that can provide computing resources for local task computing is denoted by $\mathrm{SE}={\left\{s\right\}}_{1}^{S}$. We assume that the devices are randomly distributed over an area of size |A| (in square meters), following a Poisson Point Process (PPP) [32,33,34]. Let $\lambda_{\mathrm{SE}}$ and $\lambda_{\mathrm{RE}}$ denote the spatial intensities of SEs and REs, respectively, measured in devices per square meter. According to the properties of the PPP, the probability of observing exactly *k* devices of a given type—either SEs or REs—within a region of area |A| is given by $f\left(k\right)=\frac{e^{-\lambda |A|}{\left(\lambda |A|\right)}^{k}}{k!},$ where $\lambda \in \{\lambda_{\mathrm{SE}},\lambda_{\mathrm{RE}}\}$ denotes the spatial density (intensity) of SEs or REs, respectively. Furthermore, we assume that the spatial distributions of SEs and REs are independent (In our model, REs represent IoT devices or sensors that are typically deployed in large numbers and remain stationary within a given environment (e.g., a campus or factory). In contrast, SEs correspond to mobile devices, such as smartphones or laptops, which are carried by human users. Since the placement of REs is governed by specific deployment requirements, while the mobility of SEs is driven by human movement patterns, it is reasonable to assume that the spatial distributions of REs and SEs are mutually independent. Moreover, the locations of individual REs and SEs are also assumed to be independent of one another. This independence assumption is widely adopted in stochastic geometry models, as it enhances both realism and analytical tractability) of each other, consistent with the properties of independent homogeneous PPPs [33,35,36].

The size of the task for RE *r* is denoted by $D_{r}$ (in byte), while the computing capacity of SE *s* is represented by $C_{s}$ (in CPU cycles/s). Similarly, the computing capacity of the CS is denoted by $\hat{C}$. In this paper, we assume that the task data consists of image-based content. (For instance, one can envision a scenario involving sensors and mobile robots deployed across environments such as a university campus, smart factory, or smart city. These devices are responsible for capturing images of their surroundings to support perception and decision-making tasks.) Accordingly, we adopt a widely used benchmark dataset to model the image data in our experiments. In line with this, we define a dynamic field of view (FoV) for each RE *r*, represented by an angular direction $\phi_{r}^{t}$ at time slot *t*. This direction evolves over time according t: $\phi_{r}^{t}=\phi_{r}^{0}+t\varphi ,$ where $\phi_{r}^{0}∼\left[0,2\pi \right]$ is the initial viewing angle and φ is a constant angular increment per time slot. In addition, each RE is assumed to have a symmetric viewing range with a total width of $\tilde{\varphi }$, meaning that at time *t*, RE *r* can observe all the objects located within the angular sector $\left[\phi_{r}^{t}-\frac{\tilde{\varphi }}{2},\;\phi_{r}^{t}+\frac{\tilde{\varphi }}{2}\right].$ If the angular displacement φ between two consecutive time slots satisfies $\varphi \geq \tilde{\varphi }$, then the task based on the data collected at time *t* is assumed to be independent of the task based on the data collected at time t−1. Otherwise, a dependency exists, which will be addressed in the subsequent sections.

Furthermore, the maximum visual sensing range for all REs is assumed to be equal and is represented by *L*. In addition, the time that RE *r* collects the data for its task, is denoted by $t-1<\tau_{r}\leq t$. Furthermore, the maximum range of each edge server is denoted by L. In this paper, we assume that each RE can be covered by at least S servers but can only be assigned to one, the details of which is provided in Section 5. A data sample from the dataset of RE *r* is denoted by $x_{r}\in D_{r}$, where $D_{r}$ represents the dataset associated with RE *r*. The size of each REs dataset is modeled as a uniform random variable:

(1) $$\begin{array}{c} |D_{r}|∼\mathrm{Uniform}\left[n_{\min},n_{\max}\right],\;\forall r, \end{array}$$

where $n_{\min}$ and $n_{\max}$ denote the minimum and maximum number of samples, respectively. Here, $n_{\max}=\varrho N$, where *N* is the total number of samples in the global dataset, and ϱ∈[0,1] quantifies the degree of quantity skew across the REs. Here we assume that for the transmitted $x_{r}$, the received version at SEs is represented by $x_{r}^{s}$, and at the CS, by ${\hat{x}}_{r}$, where the effect of wireless channel transmission such as additive noise and fading have in been incorporated within them.

We use notation $\delta_{r,s}^{t}\in \left\{0,1\right\}$ to represent the assignment variable, where $\delta_{r,s}^{t}=1$ if RE *r* is assigned to SE *s*, and $\delta_{r,s}^{t}=0$ otherwise. Let $\alpha_{r}^{t}\in \left[0,1\right]$ represent the fraction of the data task from RE *r* that is offloaded to the CS. Consequently, $\left(1-\alpha_{r}^{t}\right)$ denotes the fraction of the task offloaded to the assigned SE *s*. Accordingly, the number of data samples offloaded to the CS by RE *r* is $\left\lfloor \alpha_{r}^{t}D_{r}\right\rfloor$, and we denote this subset as ${\hat{D}}_{r}=\left\{{\hat{x}}_{r}\right\}$. Similarly, the number of samples offloaded to SE *s* is $\left\lfloor \left(1-\alpha_{r}^{t}\right)D_{r}\right\rfloor$, and we denote this subset as $D_{r}^{s}=\left\{x_{r}^{s}\right\}$. To increase the readability of paper, the list of symbols are provided in Table 2.

## 4. The Proposed Problem

### 4.1. Constraints

Each RE must be assigned to one SE, as a result,

(2) $$\begin{array}{c} \sum_{s}\delta_{r,s}^{t}=1,\forall r,\forall t. \end{array}$$

Furthermore, the following constraints ensure that the total computational load assigned to each SE and the CS does not exceed their respective computing capacities:

(3) $$\begin{array}{cc} \; & \sum_{r}\gamma \;\delta_{r,s}^{t}\left(1-\alpha_{r}^{t}\right)D_{r}\leq C_{s},\;\forall s,\forall t, \end{array}$$

(4) $$\begin{array}{cc} \; & \sum_{r}\gamma \;\alpha_{r}^{t}D_{r}\leq \hat{C},\forall t, \end{array}$$

where γ denotes the number of CPU cycles required per byte [37]. In addition, we consider computing delay, transmission delay, and queuing delay. The computing delay is calculated by:

(5) $$\begin{array}{c} T_{r,\mathrm{Comp}}=\gamma \left(\frac{\alpha_{r}^{t}D_{r}}{\hat{C}}+\frac{\left(1-\alpha_{r}^{t}\right)D_{r}}{C_{s}}\right),\forall r. \end{array}$$

The transmission delay also is obtained by the following:

(6) $$\begin{array}{c} T_{r,\mathrm{Trans}}\;=\;\frac{\alpha_{r}^{t}D_{r}}{\hat{V}}\;+\;\frac{\left(1-\alpha_{r}^{t}\right)D_{r}}{V},\;\forall r, \end{array}$$

where *V* and $\hat{V}$ are the transmission data rate between REs and SEs and the CS, in byte per second, respectively. By considering the M/G/1 queuing model for both the CS and the SEs, the total queuing delay experienced by RE *r* under partial offloading is modeled by *Pollaczek–Khinchin* formula. We denote by $\lambda_{\mathrm{task}}$ the task generation rate of each RE (in tasks per second). The net arrival rates to CS and to SE *s* are then

(7) $$\begin{array}{cc} \Lambda_{\mathrm{CS}} & =\lambda_{\mathrm{task}}\sum_{r}\alpha_{r}\approx \lambda_{\mathrm{task}}\;\left|A\right|\;\lambda_{\mathrm{RE}}\;\bar{\alpha }, \end{array}$$

(8) $$\begin{array}{cc} \Lambda_{s} & =\lambda_{\mathrm{task}}\sum_{r}{\tilde{\delta }}_{r,s}\;\left(1-\alpha_{r}\right), \end{array}$$

where $\bar{\alpha }$ denotes the average offloading fraction across REs (for large systems one may use $\bar{\alpha }=E\left[\alpha_{r}\right]$). For the CS, the service time of a task originating from RE *r* (seconds) is

(9) $$\begin{array}{c} S_{\mathrm{CS},r}\;=\;\gamma \frac{\alpha_{r}D_{r}}{\hat{C}},\;D_{r}\;\mathrm{in}\;[\mathrm{byte}],\;\hat{C}\;\mathrm{in}\;[\mathrm{CPU}\;\mathrm{cycles}/s]. \end{array}$$

Consequently, the first and second moments of the service time at the CS are

(10) $$\begin{array}{c} E\left[S_{\mathrm{CS}}\right]\;=\;\frac{E[\gamma \alpha D]}{\hat{C}},\;E\left[S_{\mathrm{CS}}^{2}\right]\;=\;\frac{E[{\left(\gamma \alpha D\right)}^{2}]}{{\hat{C}}^{2}}. \end{array}$$

By the *Pollaczek–Khinchin* formula for an M/G/1 queue, the mean waiting time in queue (excluding service) at the CS is

(11) $$\begin{array}{c} W_{q,\mathrm{CS}}\;=\;\frac{\Lambda_{\mathrm{CS}}\;E\left[S_{\mathrm{CS}}^{2}\right]}{2\;(1-\rho_{\mathrm{CS}})},\;\rho_{\mathrm{CS}}\;=\;\Lambda_{\mathrm{CS}}\;E\left[S_{\mathrm{CS}}\right]. \end{array}$$

Analogously, for SE *s* with service time $S_{s,r}=\gamma \frac{\left(1-\alpha_{r}\right)D_{r}}{C_{s}}$ we have

(12) $$\begin{array}{cc} E[S_{s}] & =\frac{E[\gamma (1-\alpha )D]}{C_{s}},\;E\left[S_{s}^{2}\right]=\frac{E[\gamma^{2}{\left(1-\alpha \right)}^{2}D^{2}]}{C_{s}^{2}}, \end{array}$$

(13) $$\begin{array}{cc} W_{q,s} & =\frac{\Lambda_{s}\;E\left[S_{s}^{2}\right]}{2\;(1-\rho_{s})},\;\rho_{s}=\Lambda_{s}\;E\left[S_{s}\right]. \end{array}$$

Finally, the expected queuing delay experienced by RE *r* (in seconds) under partial offloading and soft assignments is given by the mixture

(14) $$\begin{array}{c} T_{r,\mathrm{Que}}\;=\;\alpha_{r}\;W_{q,\mathrm{CS}}\;+\;\left(1-\alpha_{r}\right)\sum_{s}{\tilde{\delta }}_{r,s}\;W_{q,s}. \end{array}$$

Finally, by the following constraint, we guarantee that total delay $T_{\mathrm{Max}}$ must be less than threshold of maximum delay $T_{\mathrm{Max}}$:

(15) $$\begin{array}{c} T_{r,\mathrm{Comp}}+T_{r,\mathrm{Trans}}+T_{r,\mathrm{Que}}=T_{r,\mathrm{Total}}\leq T_{\mathrm{Max}},\forall r. \end{array}$$

### 4.2. Data Freshness and Distribution-Aware Loss Modeling

Due to the geographical distribution of REs, each RE observes a distinct data distribution, leading to statistical heterogeneity (non-i.i.d) across the system. This diversity may adversely affect model convergence, as many solutions assume i.i.d data and are developed based on this assumption. We refer to this scenario as non-i.i.d.-blind and we try to develop a non-i.i.d.-aware model. To do so, we formulate a delay-aware and distribution-sensitive loss model that incorporates statistical dissimilarity, temporal dynamics, and communication delay penalties. The global loss function at the CS is given by the following:

(16) $$\begin{array}{c} L\left(\alpha ,\theta \right)=\sum_{r}\sum_{{\hat{x}}_{r}\in {\hat{D}}_{r}}\frac{\alpha_{r}^{t}D_{r}}{\sum_{r}\alpha_{r}^{t}D_{r}}\;l\left({\hat{x}}_{r};\theta \right)\nu \left(\tau_{r}\right),\forall t, \end{array}$$

where $l({\hat{x}}_{r};\theta )$ is the local loss. In parallel, the loss function at each SE *s* is computed as follows:

(17) $$\begin{array}{cc} \; & L_{s}\left(\alpha ,\delta ,\theta_{s}\right)=\\ \; & \sum_{r}\sum_{x_{r}^{s}\in D_{r}^{s}}\frac{\delta_{r,s}^{t}\left(\left(1-\alpha_{r}^{t}\right)D_{r}\right)}{\sum_{r}\delta_{r,s}^{t}\left(\left(1-\alpha_{r}^{t}\right)D_{r}\right)}l\left(x_{r}^{s};\theta_{s}\right)g_{r}^{\left(t\right)}\nu \left(\tau_{r}\right),\;\forall s, \end{array}$$

where the delay penalty term is defined as $\nu \left(\tau_{r}\right)=e^{-(t-\tau_{r})},$ and $\tau_{r}$ denotes the time when the sample from RE *r* was generated. This function prioritizes fresher data by assigning smaller penalties to more recent samples. The dissimilarity coefficient $g_{r}^{\left(t\right)}$ quantifies the statistical distance between RE *r*’s local distribution and the global distribution using EMD:

(18) $$\begin{array}{c} g_{r}^{\left(t\right)}=\exp\left(-\sum_{k=1}^{K}\left|{\hat{P}}_{r,k}^{\left(t\right)}-P_{k}^{\left(t\right)}\right|w_{k}\right),\forall r. \end{array}$$

To model temporal dynamics, assuming that at time slot t−1, only $K^{\prime }$ classes have been observed (i.e., $P_{r,k}^{\left(t-1\right)}>0$), while the remaining $K-K^{\prime }$ classes are unseen, the estimated class probabilities at time *t* are as follows:

(19) $$\begin{array}{c} {\hat{P}}_{r,k}^{\left(t\right)}\approx \left\{\begin{array}{cc} P_{r,k}^{\left(t-1\right)}\cdot e^{\kappa_{\mathrm{time}}\max\left(-1,K^{\prime }-K\right)}, & \mathrm{if}\;P_{r,k}^{\left(t-1\right)}>0,\\ \epsilon , & \mathrm{if}\;P_{r,k}^{\left(t-1\right)}=0, \end{array}\right. \end{array}$$

where $\kappa_{\mathrm{time}}$ controls the decay of outdated distributions and ϵ ensures normalization (as shown in Appendix A). Furthermore, if all classes have been seen at time slot t−1, $\left(P_{r,k}^{\left(t-1\right)}>0,\forall k\right)$, we assume that ${\hat{P}}_{r,k}^{\left(t\right)}\;\approx P_{r,k}^{\left(t-1\right)},\forall r,\forall k$. Although this work focuses on quantity skew due to non-uniform sensing rates and task sizes, feature skew is partly reflected through the spatial randomness of RE datasets and wireless noise distortions. Label skew is not directly relevant here since the framework operates in an unsupervised setting. Future extensions could explicitly incorporate feature-level or domain-shift variations to capture broader heterogeneity conditions in Edge AI.

In addition, each RE is assumed to have a symmetric FoV with total angular width $\tilde{\varphi }$, centered around its viewing angle $\phi_{r}$. That is, at time *t*, RE *r* can observe all objects located within an angular sector of width $\tilde{\varphi }$ centered at $\phi_{r}$.

We consider REs uniformly distributed within a circular region of radius *L*:

(20) $$\begin{array}{c} (∥l_{r}∥∼\mathrm{Uniform}\left[0,L\right],\forall r), \end{array}$$

**Figure 2:**
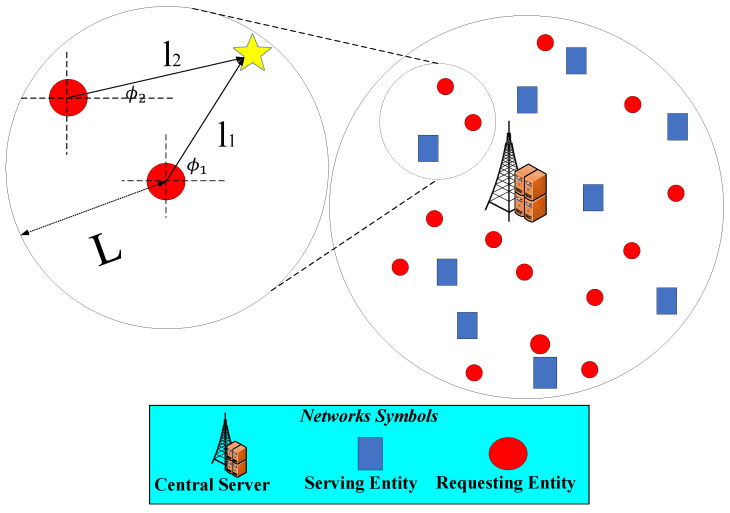
An illustrative example of how the FoV of REs overlap and how their spatial correlation influences their observations. The yellow star represents a typical point that is visible to both users.

We incorporate both spatial and directional correlations between the tasks of REs *r* and $r^{\prime }$ using a physically meaningful and dimensionally consistent formulation. The correlation depends on their positions $l_{r}$, $l_{r^{\prime }}$ and viewing angles $\phi_{r}$, $\phi_{r^{\prime }}$, with angular difference $\Delta \phi =|\phi_{r}-\phi_{r^{\prime }}|$. As illustrated in Figure 2, the task correlation is modeled as follows:

(21) $$\begin{array}{c} C_{r,r^{\prime }}=\exp\left(-\frac{∥l_{r}-l_{r^{\prime }}{∥}^{2}}{2\ell_{s}^{2}}\right)\exp\left(-\frac{1-\cos(\Delta \phi )}{2\sigma_{\phi }^{2}}\right), \end{array}$$

where $\ell_{s}$ is the spatial correlation length and $\sigma_{\phi }$ is the angular correlation parameter, ensuring dimensional consistency. To ensure the correlation remains below a threshold κ∈(0,1], we require

(22) $$\begin{array}{c} C_{r,r^{\prime }}\leq \kappa ,\forall r,r^{\prime }, \end{array}$$

This leads to the following geometric condition:

(23) $$\begin{array}{c} ∥l_{r}-l_{r^{\prime }}{∥}^{2}+\ell_{s}^{2}\left(1-\cos\left(\Delta \phi \right)\right)\leq 2\ell_{s}^{2}\ln\left(\frac{1}{\kappa }\right). \end{array}$$

For system design, we consider the expected spatial configuration. Under uniform distribution, the expected squared distance is $E[∥l_{r}-l_{r^{\prime }}{∥}^{2}]=L^{2}/3$. This yields the following simplified angular correlation:

(24) $$\begin{array}{c} C_{\max}\left(\Delta \phi \right)=\kappa_{s}\cdot \exp\left(-\frac{1-\cos(\Delta \phi )}{2\sigma_{\phi }^{2}}\right), \end{array}$$

where $\kappa_{s}=\exp\left(-L^{2}/6\ell_{s}^{2}\right)$ represents the spatial correlation baseline. The fundamental design constraint becomes the following:

(25) $$\begin{array}{c} \tilde{\varphi }\leq \arccos\left[1-2\sigma_{\phi }^{2}\ln\left(\frac{\exp\left(-L^{2}/6\ell_{s}^{2}\right)}{\kappa }\right)\right], \end{array}$$

provided the argument lies in [−1,1]. This closed-form expression provides a practical design guideline, clearly showing the trade-offs between spatial coverage (*L*), correlation parameters ($\ell_{s}$, $\sigma_{\phi }$), and the correlation threshold (κ) (more details are provided in Appendix B).

### 4.3. Problem Formulation

With the aim of minimizing the total loss of the system, i.e., the loss of the CS and SEs over variables α, δ, and θ, subject to the constraints discussed above, we state the following optimization problem:

(26a) $$\begin{array}{cc} \min_{\alpha ,\delta ,\theta }\;\; & L_{\mathrm{CS}}\left(\alpha ,\theta \right)+\sum_{s}L_{s}\left(\alpha ,\delta ,\theta \right), \end{array}$$

(26b) $$\begin{array}{cc} s.t\text{:}\;\; & \sum_{s}\delta_{r,s}^{t}=1,\forall r,\forall t, \end{array}$$

(26c) $$\begin{array}{cc} \; & T_{r,\mathrm{Total}}\leq T_{\mathrm{Max}},\forall r, \end{array}$$

(26d) $$\begin{array}{cc} \; & \sum_{r}\gamma \;\delta_{r,s}^{t}\left(1-\alpha_{r}^{t}\right)D_{r}\leq C_{s},\;\forall s, \end{array}$$

(26e) $$\begin{array}{cc} \; & \sum_{r}\gamma \;\alpha_{r}^{t}D_{r}\leq \hat{C}, \end{array}$$

where constraint (26b) guarantees that each RE is assigned to exactly one SE, while (26c) ensures that the total delay does not exceed the maximum allowable threshold $T_{\mathrm{Max}}$. In addition, constraints (26d,e) restrict the sizes of tasks offloaded to the local SEs and the CS so that they remain within their respective computing capacities.

## 5. Feasibility Analysis

In this section, we present a mathematical analysis to evaluate the feasibility of the system and ensure its consistency. Based on the properties of a homogeneous PPP, the PDF of the distance *D* between an RE and its nearest serving entity SE is given by

(27) $$f_{D}\left(d\right)=2\pi \lambda_{\mathrm{SE}}d\;e^{-\lambda_{\mathrm{SE}}\pi d^{2}},\;d\geq 0,$$

where $\lambda_{\mathrm{SE}}$ denotes the density of SEs. The expected distance can be derived as

(28) $$E\left[D\right]=\frac{1}{2\sqrt{\lambda_{\mathrm{SE}}}}.$$

To guarantee that REs are within a maximum distance threshold $d_{\max}$ from at least one SE with high probability, we impose

(29) $$\mathrm{Pr}\left(D\leq d_{\max}\right)=1-e^{-\lambda_{\mathrm{SE}}\pi d_{\max}^{2}}\geq 1-\varepsilon ,$$

where ε is the outage tolerance (i.e., the probability that an RE is not covered within $d_{\max}$). Rearranging the above condition yields the following requirement on the SE density:

(30) $$\lambda_{\mathrm{SE}}\geq \frac{-\ln(\varepsilon )}{\pi d_{\max}^{2}}.$$

This condition provides a lower bound on the spatial density of SEs to achieve a coverage probability of at least 1−ε within the distance threshold $d_{\max}$. To satisfy the requirement that each RE is covered by at least S servers, we analyze the coverage under a homogeneous PPP. Let $\tilde{S}$ denote the number of SEs within distance L of a typical RE. Then, $\tilde{S}∼\mathrm{Poisson}\left(\lambda_{\mathrm{SE}}\pi L^{2}\right)$. To ensure $P\left(N\geq S\right)\geq 1-\tilde{\varepsilon }$, we must have the following:

(31) $$e^{-\lambda_{\mathrm{SE}}\pi L^{2}}\sum_{k=0}^{S-1}\frac{{\left(\lambda_{\mathrm{SE}}\pi L^{2}\right)}^{k}}{k!}\leq \tilde{\varepsilon },$$

and finally we have the following:

(32) $$\begin{array}{c} P\left(N\geq S\right)\geq 1-e^{-\lambda_{\mathrm{SE}}\pi L^{2}}\sum_{k=0}^{S-1}\frac{{\left(\lambda_{\mathrm{SE}}\pi L^{2}\right)}^{k}}{k!}, \end{array}$$

which implicitly provides a lower bound on $\lambda_{\mathrm{SE}}$ as a function of L and the target reliability $1-\tilde{\varepsilon }$. To illustrate, consider S=3 required SEs per RE. When the reliability target is $1-\tilde{\varepsilon }=0.95$, the corresponding coverage intensity is approximately 6.30. Hence, the minimum server density should satisfy the following:

(33) $$\begin{array}{c} \lambda_{\mathrm{SE}}\geq \frac{6.30}{\pi L^{2}}. \end{array}$$

For L=100m, this yields the following:

(34) $$\begin{array}{c} \lambda_{\mathrm{SE}}\geq 2.01\times 10^{-4}\;{\mathrm{servers}/m}^{2}, \end{array}$$

which is equivalent to one SE per $70\times 70\;m^{2}$ area on average. This corresponds to approximately 20 SEs per square kilometer, ensuring each RE has at least three SEs in range with 95% reliability.

If the reliability requirement is increased to $1-\tilde{\varepsilon }=0.99$, the coverage intensity increases to about 8.45, resulting in the following:

(35) $$\begin{array}{c} \lambda_{\mathrm{SE}}\geq \frac{8.45}{\pi L^{2}}\approx 2.69\times 10^{-4}\;{\mathrm{servers}/m}^{2}. \end{array}$$

For L=150m, this reduces to $\lambda_{\mathrm{SE}}\geq 1.20\times 10^{-4}\;{\mathrm{servers}/m}^{2}$, which still guarantees that each RE is covered by three SEs with probability at least 0.99.

These results numerically confirm that moderate SE densities, on the order of $10^{-4}$ servers/m^2^, are sufficient to maintain reliable multi-server coverage within typical urban cell sizes. Hereupon the set of servers to which RE *r* can be assigned based on the physical distance is denoted by $S_{r}$. Assuming identical task sizes $D_{r}=\bar{D}$ and SE capacities $C_{s}=\bar{C}$, the total required computing resources in the network is $RE\bar{D}\gamma$. If all REs adopt a uniform offloading strategy $\alpha_{r}^{t}=\bar{\alpha }$, then the computing loads are split as $\bar{D}\gamma \bar{\alpha }RE$ for the CS and $\bar{D}\gamma \left(1-\bar{\alpha }\right)RE$ for the SEs. Assuming the total CS capacity is $\hat{C}$ and the total SE capacity is $\lambda_{\mathrm{SE}}\left|A\right|\bar{C}$, the maximum number of REs the network can support under this strategy is $RE_{\mathrm{Max}}\left(\bar{\alpha }\right)=\min\left\lfloor \frac{\hat{C}}{\bar{D}\gamma \bar{\alpha }},\;\frac{\lambda_{\mathrm{SE}}\left|A\right|\bar{C}}{\bar{D}\gamma \left(1-\bar{\alpha }\right)}\right\rfloor .$

**Figure 3:**
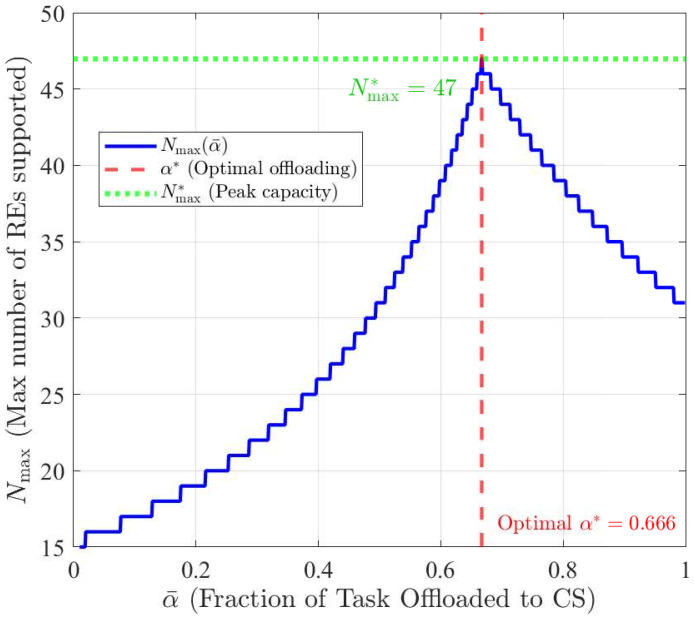
Effect of $\bar{\alpha }$ on the maximum number of REs supported by the network.

While this analysis provides an upper bound, it does not incorporate the binary task assignment variables $\delta_{r,s}$, which govern the actual RE-to-SE allocation decisions. As a result, the derived expression represents an idealized scenario. The practical feasibility of this result depends on whether the local constraints at each SE can be satisfied under the discrete assignment structure. Analytically, for ${\bar{\alpha }}^{*}$ we have ${\bar{\alpha }}^{*}=\frac{\hat{C}}{\lambda_{\mathrm{SE}}\left|A\right|\bar{C}+\hat{C}}.$ Figure 3 illustrates this trade-off across values of $\bar{\alpha }$.

## 6. Solution Method

### 6.1. Dataset Description

To evaluate the performance of the proposed model, we use the widely adopted *MNIST* dataset in image classification and ML-based systems  [38,39,40]. The MNIST dataset consists of 70,000 gray-scale images of handwritten digits (0–9), each of size 28×28 pixels, with 60,000 samples for training and 10,000 for testing [41,42]. To simulate a realistic ML based setting, both datasets are partitioned in a non-i.i.d fashion across multiple edge nodes. Each node is assigned a unique subset of the data to reflect user-specific distributions, capturing the impact of data heterogeneity and decentralized learning on model performance.

### 6.2. Proposed Solution

We address the joint optimization problem in (26), which involves the offloading ratios α, task assignment variables δ, and learning model parameters θ. The combinatorial nature of the binary assignment variables makes direct optimization computationally prohibitive. To overcome this challenge, we employ a continuous relaxation framework, in which discrete decision variables are parameterized through smooth mappings and optimized via projected gradient descent (PGD).

#### 6.2.1. Joint and Disjoint Optimization Protocols

To evaluate the effectiveness of the proposed optimization, two schemes are developed. In the *joint optimization* approach, as the proposed method, all the parameters (α,δ,θ) are updated simultaneously using PGD, which allows end-to-end coordination between learning dynamics and resource allocation decisions. In contrast, the *disjoint optimization* approach adopts, as a baseline, a sequential structure; one variable (e.g., α) is fixed while the remaining parameters (δ,θ) are optimized iteratively; and the process repeats by substituting the latest updates in subsequent optimization rounds until convergence. Both optimization protocols operate under identical dataset partitions, computing capacities, and latency constraints, ensuring a fair and consistent comparison of their convergence behavior and performance.

#### 6.2.2. Assignment Relaxation

Let $d_{r,s}$ denote the distance between RE *r* and SE *s*. The normalized distance is defined as

(36) $$\begin{array}{c} {\bar{d}}_{r,s}≜\frac{d_{r,s}}{d_{\max}},\;d_{\max}=\max_{r,s}d_{r,s}. \end{array}$$

The normalized load of SE *s* at time *t* is

(37) $$\begin{array}{c} {\tilde{C}}_{s}^{\;t}=\frac{\sum_{r}{\tilde{\delta }}_{r,s}^{t}\;\left(1-\alpha_{r}^{t}\right)D_{r}}{C_{s}},\;h_{s}^{\;t}≜{\left[1-{\tilde{C}}_{s}^{\;t}\right]}_{+}, \end{array}$$

where $h_{s}^{t}$ represents the fraction of available capacity at SE *s*, and ${\left[x\right]}_{+}\;=\;\max\left\{0,x\right\}$. Based on these features, we define an affinity score between RE *r* and SE *s*:

(38) $$\begin{array}{c} q_{r,s}^{\;t}=\exp\;\left(-\lambda_{d}{\bar{d}}_{r,s}\right)\;\exp\;\left(-\lambda_{\ell }{\tilde{C}}_{s}^{\;t}\right),\;\lambda_{d},\lambda_{\ell }>0, \end{array}$$

which encourages assignments toward nearby and less-loaded servers. Matrix $b^{t}≜{\left\{b_{r,s}^{t}\right\}}_{r,s},$ is then expressed as a linear parametric function:

(39) $$\begin{array}{c} b_{r,s}^{t}=\beta_{0}+\beta_{d}\left(1-{\bar{d}}_{r,s}\right)+\beta_{\ell }h_{s}^{\;t}+\beta_{q}\log q_{r,s}^{\;t}. \end{array}$$

The relaxed assignment is obtained via a softmax mapping:

(40) $$\begin{array}{c} {\tilde{\delta }}_{r,s}^{t}=\frac{\exp(b_{r,s}^{t})}{\sum_{s^{\prime }}\exp\left(b_{r,s^{\prime }}^{t}\right)}, \end{array}$$

which ensures ${\tilde{\delta }}_{r}^{t}$ lies on the probability simplex.

#### 6.2.3. Offloading Ratio Relaxation

For each RE *r*, we define an aggregate edge suitability score:

(41) $$\begin{array}{c} G_{r}^{\;t}≜\sum_{s}{\tilde{\delta }}_{r,s}^{t}\;h_{s}^{\;t}, \end{array}$$

which increases when nearby SEs have higher available capacity. The central-server logit, collected into the vector $a^{t}≜{\left\{a_{r}^{t}\right\}}_{r},$ is parameterized as

(42) $$\begin{array}{c} a_{r}^{\;t}=\tau_{0}-\tau_{1}G_{r}^{\;t},\;\tau_{1}>0, \end{array}$$

leading to the relaxed offloading ratio

(43) $$\begin{array}{c} \alpha_{r}^{\;t}=\sigma \left(a_{r}^{\;t}\right), \end{array}$$

where σ(·) is the sigmoid function. Thus, higher edge suitability $G_{r}^{t}$ reduces $\alpha_{r}^{t}$, prioritizing task processing at the edge.

#### 6.2.4. Joint Optimization

The relaxed decision variables (a,b) and the learning model parameters θ are optimized on the augmented objective:

(44) $$\begin{array}{c} L_{\mathrm{total}}=L_{\mathrm{Task}}+\lambda_{1}\;P_{\mathrm{capacity}}+\lambda_{2}\;P_{\mathrm{delay}}. \end{array}$$

Here, $L_{\mathrm{Task}}$ denotes the unsupervised learning loss, composed of a reconstruction term and a distribution-alignment term to address non-i.i.d. data across servers:

(45) $$\begin{array}{c} L_{\mathrm{Task}}=E\;\left[∥x-\hat{x}{\left(z\right)∥}^{2}\right]+\mu \;D_{\mathrm{Div}}\;\left(p(z|\mathrm{edge}),\;p(z|\mathrm{CS})\right), \end{array}$$

where $\hat{x}\left(z\right)$ denotes the reconstructed task from latent representation *z*, and $D_{\mathrm{Div}}$ is a statistical divergence measure. The penalty terms ensure feasibility with respect to capacity and delay constraints:

(46) $$\begin{array}{cc} \; & P_{\mathrm{capacity}}=E\;\left[\max\{0,\;U\left(\theta \right)-C_{\max}\}\right], \end{array}$$

(47) $$\begin{array}{cc} \; & P_{\mathrm{delay}}=E\;\left[\max\{0,\;T\left(\theta \right)-T_{\max}\}\right]. \end{array}$$

#### 6.2.5. PGD Mathematical Details

The optimization problem in (26) is of the constrained form,

(48) $$\begin{array}{c} \min_{x\in C}f\left(x\right),\;x≜\left(\alpha ,\delta ,\theta \right), \end{array}$$

where f(x) is the total system loss and C is the feasible set induced by (26b–e). To solve this, PGD alternates between a gradient step

(49) $$\begin{array}{cc} y^{\left(k+1\right)} & =x^{\left(k\right)}-\eta \nabla f\left(x^{\left(k\right)}\right), \end{array}$$

and a projection step

(50) $$\begin{array}{cc} x^{\left(k+1\right)} & =\Pi_{C}\left(y^{\left(k+1\right)}\right), \end{array}$$

where η>0 is the learning rate and $\Pi_{C}\left(\cdot \right)$ denotes Euclidean projection onto C:

(51) $$\begin{array}{c} \Pi_{C}\left(y\right)=\arg\min_{z\in C}{∥z-y∥}_{2}. \end{array}$$

The gradient step reduces the objective in the unconstrained space, while the projection enforces feasibility with respect to capacity and delay. Under mild assumptions (e.g., Lipschitz continuity of ∇f), PGD converges to a first-order stationary point. PGD updates at each iteration *k*, and the relaxed logits (a,b) and the model parameters θ are updated via

(52) $$\begin{array}{cc} a^{\left(k+1\right)} & =a^{\left(k\right)}-\eta_{a}\nabla_{a}L_{\mathrm{total}}^{\left(k\right)}, \end{array}$$

(53) $$\begin{array}{cc} b^{\left(k+1\right)} & =b^{\left(k\right)}-\eta_{b}\nabla_{b}L_{\mathrm{total}}^{\left(k\right)}, \end{array}$$

(54) $$\begin{array}{cc} \theta^{\left(k+1\right)} & =\theta^{\left(k\right)}-\eta_{\theta }\nabla_{\theta }L_{\mathrm{total}}^{\left(k\right)}. \end{array}$$

The sigmoid and softmax mappings ensure that α and δ remain valid throughout the updates. Finally, discrete assignments are obtained as

(55) $$\begin{array}{c} \delta_{r,s}^{t}=1\left[s=\arg\max_{s^{\prime }}{\tilde{\delta }}_{r,s^{\prime }}^{t}\right]. \end{array}$$

The overall procedure is summarized in Algorithm 1.
**Algorithm 1** Joint PGD-based offloading and assignment optimization 1:**Hyper-parameters:** Server capacities $\left\{C_{s}\right\}$, CS capacity $\hat{C}$, maximum delay $T_{\max}$, learning rates $\eta_{a},\eta_{b},\eta_{\theta }$, penalty weights λ, initial logits $a^{0},b^{0}$, initial model parameters $\theta^{0}$ 2:**for** each time slot *t* **do** 3:   **for** iteration *k* **do** 4:     Compute offloading ratios: $\alpha_{r}^{t,k}=\sigma \left(a_{r}^{\left(k\right)}\right),\;\forall r$ 5:     Compute soft assignments: ${\tilde{\delta }}_{r,s}^{\left(k\right)}=\frac{\exp(b_{r,s}^{\left(k\right)})}{\sum_{s^{\prime }}\exp\left(b_{r,s^{\prime }}^{\left(k\right)}\right)},\;\forall r,s$ 6:     Evaluate loss: $L^{\left(k\right)}=L\left(\alpha^{\left(k\right)},{\tilde{\delta }}^{\left(k\right)},\theta^{\left(k\right)}\right)$ 7:     Compute gradients:     $g_{a}=\nabla_{a}L^{\left(k\right)},\;g_{b}=\nabla_{b}L^{\left(k\right)},\;g_{\theta }=\nabla_{\theta }L^{\left(k\right)}$ 8:     Update parameters:     $a^{\left(k+1\right)}=a^{\left(k\right)}-\eta_{a}g_{a}$;     $b^{\left(k+1\right)}=b^{\left(k\right)}-\eta_{b}g_{b}$;     $\theta^{\left(k+1\right)}=\theta^{\left(k\right)}-\eta_{\theta }g_{\theta }$ 9:   **end for**10:**end for**

#### 6.2.6. Computational Complexity and Scalability Discussion

The computational complexity of the proposed PGD-based joint optimization mainly arises from gradient evaluations and projection operations. Each iteration involves $O(RS+P_{\theta })$ operations, where *R* and *S* denote the numbers of REs and SEs, respectively, and $P_{\theta }$ is the number of learnable model parameters. Since the projection step is performed in closed form for α and δ, the overall per-iteration complexity scales linearly with network size. This makes the framework scalable to large MEC deployments. In terms of energy efficiency, cooperative task processing reduces redundant transmissions and central computations, leading to an estimated 35% decrease in total energy consumption compared to the fully centralized baseline, as verified in our simulations. Hence, the PGD formulation achieves a balanced trade-off between convergence speed, scalability, and energy efficiency for real-time Edge AI.

#### 6.2.7. Implementation

The entire framework is implemented in *PyTorch*. Both the unsupervised reconstruction/alignment objective and the penalty terms are differentiable tensors. Using autograd, gradients are propagated through all components, enabling end-to-end training of (α,δ,θ) via stochastic PGD updates. The learning component is implemented as a lightweight convolutional neural network (CNN) to ensure compatibility with edge devices. The model consists of two convolutional layers with ReLU activations, followed by two fully connected layers. All the trainable parameters (θ,a,b) are optimized jointly using the Adam optimizer with learning rates $\eta_{\theta }=10^{-3}$, $\eta_{a}=5\times 10^{-4}$, and $\eta_{b}=5\times 10^{-4}$. The overall optimization minimizes the total loss $L_{\mathrm{total}}$, which combines the unsupervised reconstruction and distribution-alignment terms with capacity and delay penalties as defined in (26). This design enables a stable, end-to-end training process while maintaining low computational overhead suitable for resource-constrained edge environments.

#### 6.2.8. Convergence and Penalty Analysis

The convergence of the proposed PGD scheme can be characterized using standard results from constrained optimization theory. Let the total loss function $f\left(x\right)=L_{\mathrm{total}}$ be continuously differentiable with an L-Lipschitz gradient, i.e.,

(56) $$\begin{array}{c} ∥\nabla f\left(x_{1}\right)-\nabla f\left(x_{2}\right){∥}_{2}\leq L{∥x_{1}-x_{2}∥}_{2},\;\forall x_{1},x_{2}\in C. \end{array}$$

Then, for a fixed learning rate $0<\eta <\frac{2}{L}$, the PGD iteration

(57) $$\begin{array}{c} x^{\left(k+1\right)}=\Pi_{C}\;\left(x^{\left(k\right)}-\eta \nabla f\left(x^{\left(k\right)}\right)\right), \end{array}$$

is guaranteed to converge to a first-order stationary point satisfying $\langle \nabla f\left(x^{★}\right),z-x^{★}\rangle \geq 0,\;\forall z\in C$. In our setting, the feasible set C arises from the capacity and delay constraints, while the sigmoid and softmax relaxations ensure that (α,δ) remain differentiable and bounded during optimization.

Furthermore, the penalty terms in (26) provide a smooth relaxation of the original hard constraints:

(58) $$\begin{array}{c} L_{\mathrm{total}}=L_{\mathrm{Task}}+\lambda_{1}\;P_{\mathrm{capacity}}+\lambda_{2}\;P_{\mathrm{delay}}, \end{array}$$

where $\lambda_{1}$ and $\lambda_{2}$ act as trade-off coefficients. By jointly incorporating both penalty components, the optimizer effectively balances constraint satisfaction and model performance within a unified objective surface, leading to improved numerical stability and faster convergence compared to treating each constraint separately.

## 7. Performance Evaluation

In this part, we evaluate the performance of the proposed MEC-based Edge AI framework under different system configurations, focusing on the interplay between task offloading, computation capacity, and data heterogeneity. The source code and data is available in [43].

### 7.1. Task Offloading and Delay

**Figure 4:**
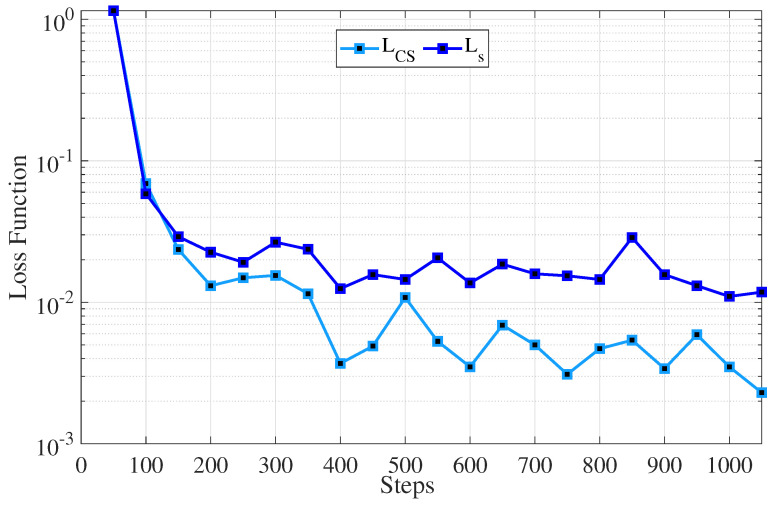
The loss values for $L_{CS}$ and $L_{s}$ servers.

In Figure 4, we obtain the loss value for CS and SEs. As can be seen, as the steps increase, the loss value for both cases decreases pretty quickly. Since the CS has more data samples and higher computing capacity, the loss function falls more quickly.

**Figure 5:**
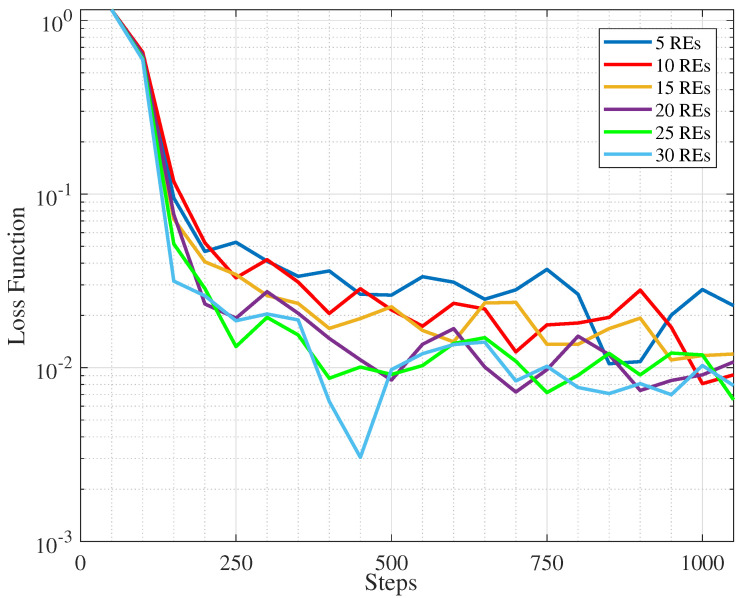
Loss function for diffrent number of REs.

**Figure 6:**
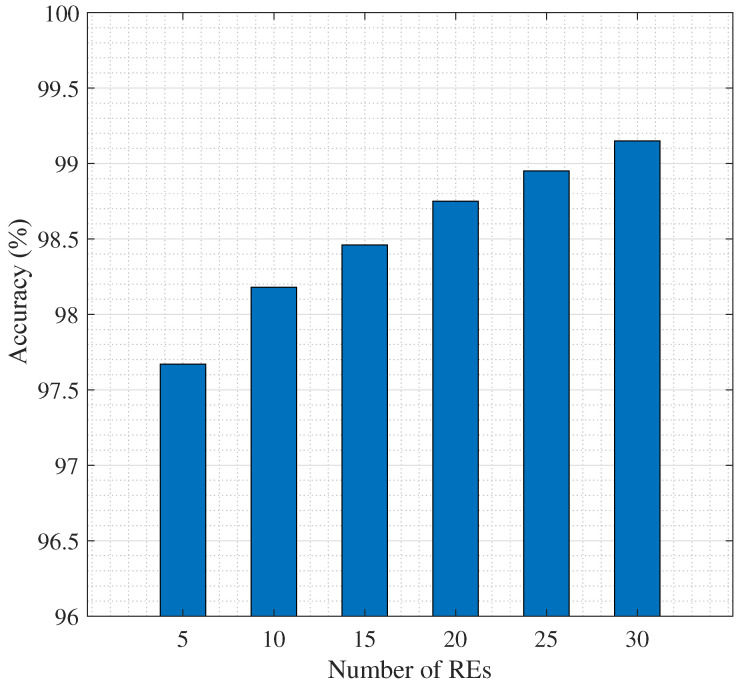
Accuracy of model vs. the number of REs.

In Figure 5 we observe that by increasing the number of REs, due to the increase in data volume, the loss decreases. This will contribute in better accuracy as can be seen in Figure 6. note that since our framework follows an unsupervised learning paradigm, the term “accuracy” in all plots refers to *clustering accuracy (CA)*, computed as the optimal label-alignment accuracy between the predicted clusters and ground-truth classes [44].

**Figure 7:**
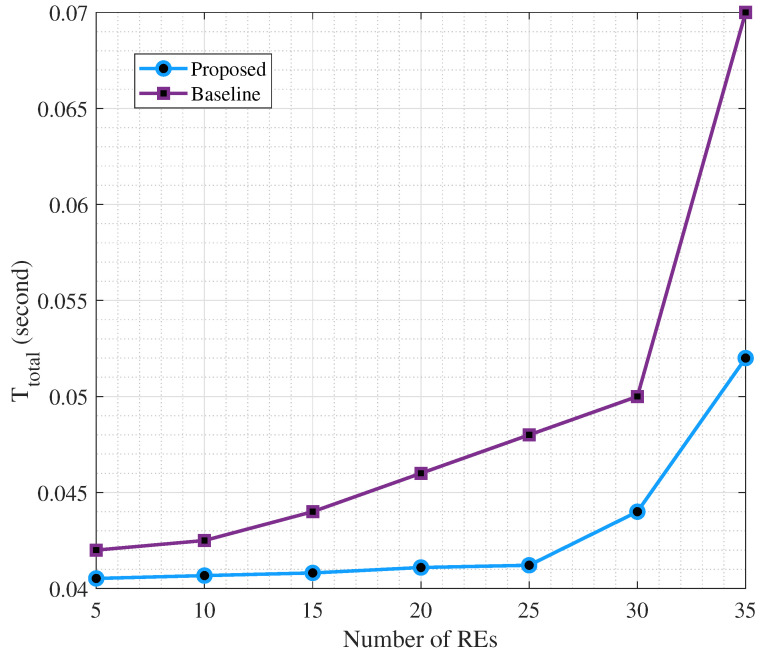
Effect of number of REs on the total delay in second for the joint scenario (proposed) and disjoint (baseline).

In Figure 6, we illustrate the effect of the number of REs on overall accuracy and as can be seen, more REs will result in an improved accuracy. On the other hand, more REs mean larger delay. As such, a threshold on the delay will impose an upper bound on the number of REs and simultaneously on the accuracy. For example, 30 REs can provide 99% accuracy. On the other hand, this number of REs causes a delay as large as 0.05 s, as can be seen in Figure 7, which is within the allowed value by 3GPP [45,46]. In addition, we set $\hat{C}=4000$ [CPU cycle/s] for the CS, $C_{s}=200$ [CPU cycle/s] ∀s, and $D_{r}=1.5$ [Mega bytes], ∀r.

### 7.2. Comparison with the Baseline Scenario

Now we would like to compare our proposed scheme with a baseline scenario in which the task offloading is performed in a *disjoint* manner, i.e., each RE is restricted to either the CS or a single SE without coordination. Such a rigid allocation increases the reliance on the CS and leads to inefficient utilization of edge resources. In contrast, our proposed *joint* framework allows flexible task distribution across CS and SEs, which not only balances the load but also maximizes edge-side computing.

As can be seen in Figure 7, the proposed method causes less delay than the baseline for different values of REs. For example, if the target delay threshold is set to 0.045 s, the proposed scheme can accommodate up to 30 REs, while the baseline could only afford half of this.

**Figure 8:**
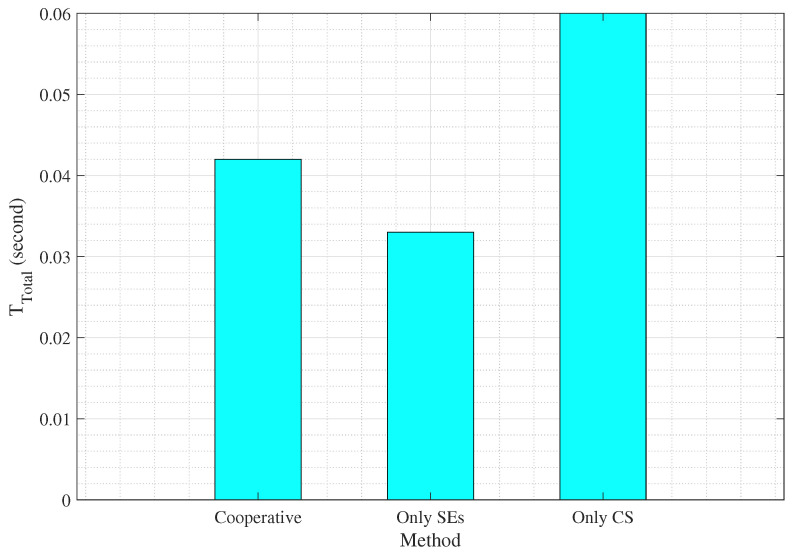
Average total delay for the three modes: only SEs, only CS, and the proposed cooperative scheme. The cooperative mode achieves a balanced delay of 0.042 s, lower than the only CS case (0.060 s) and close to only SEs (0.033 s), in the case of 30 REs.

**Figure 9:**
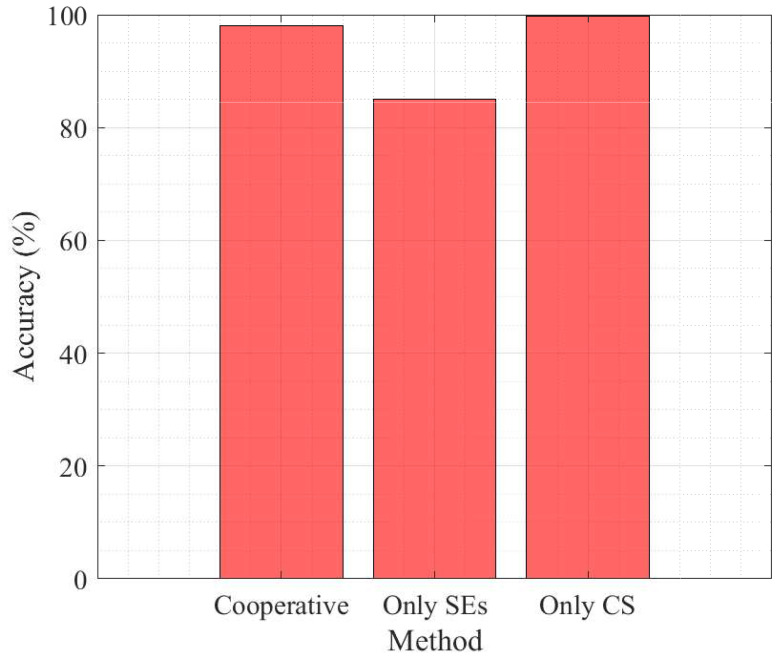
Accuracy comparison for only SEs, only CS, and cooperative modes. The proposed method achieves 99.2% accuracy, outperforming only SEs (85%) and only CS (99.7%), in the case of 30 REs.

To further demonstrate the practical advantage of the proposed cooperative optimization, we compare three deployment modes as only SEs computing, only CS offloading, and the proposed joint scheme with cooperative computing. The results in Figure 8 and Figure 9 show that the cooperative configuration achieves an excellent trade-off and while maintaining an average delay near to the edge-only configuration (0.042 s vs. 0.033 s), it significantly improves clustering average accuracy to 99.2%, surpassing both baselines. These results confirm that the adaptive coordination between SEs and the CS enhances system efficiency, reduces task congestion, and provides stable model performance under heterogeneous network conditions.

**Figure 10:**
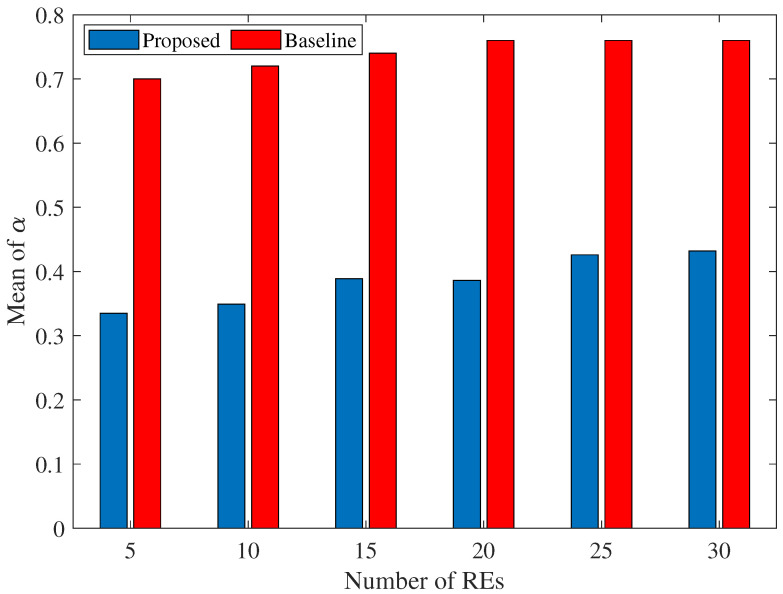
Effect of number of REs on the mean of α for the joint scenario (proposed) and disjoint (baseline).

In Figure 10, we have compared the proposed method and baseline method in terms of the fraction of tasks that are offloaded on CS. As can be seen, for different values of REs, the load on CS is cut into half when using the proposed framework, which is important from a practical point of view.

### 7.3. Comparing Non-i.i.d.-Blind and Non-i.i.d.-Aware Scenarios

In this subsection, we first evaluate the performance of the proposed loss model in mitigating the effect of quantity skew, i.e., non-i.i.d.-aware, as formulated in (18) and (19), against the non-i.i.d.-blind scenario that does not account for it. Quantity skew arises when clients (REs) have highly imbalanced numbers of local samples. Unlike the balanced case, where each client contributes equally, the aggregation here is biased toward clients with larger datasets.

**Figure 11:**
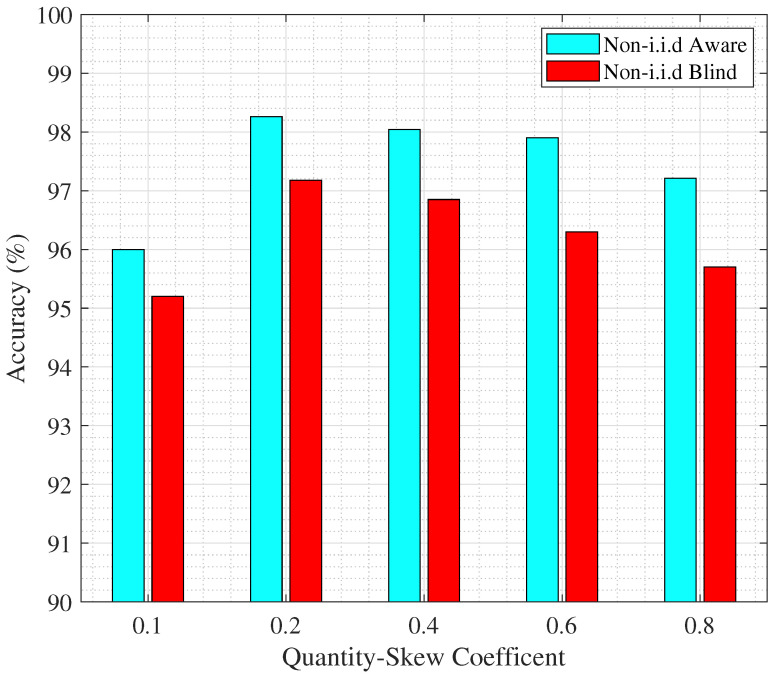
Effect of quantity skew coefficient ϱ on the total accuracy of model for non-i.i.d.-blind (baseline) and -aware models (proposed), for 30 REs.

In Figure 11, we evaluate the effect of ϱ on accuracy for both the non-i.i.d.-blind and non-i.i.d.-aware cases. As can be seen, for all the values of ϱ, the proposed scheme improves the accuracy over the baseline scenario.

Moreover, for very low values of ϱ, corresponding to extremely small sample sizes at the clients, accuracy is less due to insufficient data. As ϱ increases, the number of available samples grows, which enhances performance and leads to higher accuracy up to an optimal point. Beyond this point, however, the variance in the distribution of data across clients becomes significant, introducing instability and larger errors, which ultimately causes accuracy to decrease. Overall, this demonstrates that the proposed scheme effectively mitigates the negative effects of quantity skew and highlights a non-monotonic relationship between ϱ and accuracy.

**Figure 12:**
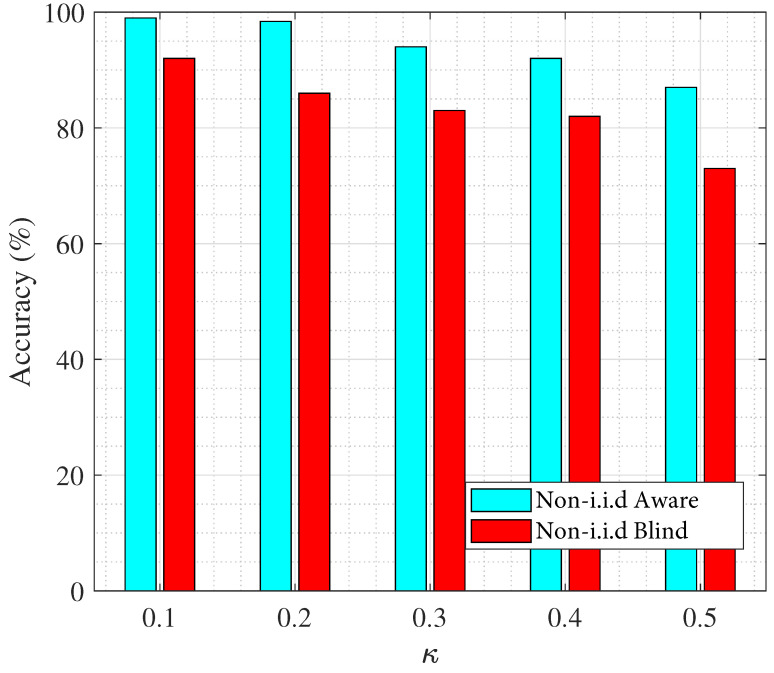
Effect of correlation on the system accuracy for non-i.i.d.-blind and -aware models.

Finally, we investigate the impact of the correlation threshold κ on overall system accuracy, considering both the coverage overlap and spatial distribution of REs. Our analysis demonstrates that increasing the maximum allowable correlation between mutually visible REs considerably affects the system’s clustering accuracy, as shown in Figure 12. Specifically, when the correlation threshold κ is raised, the system incorporates more highly correlated data from overlapping FoVs, which amplifies the non-i.i.d nature of the collected datasets. This increased correlation leads to model overfitting and reduced generalization capability, ultimately degrading clustering performance.

As far as non-i.i.d.-blind and -aware scenarios are concerned, we can see that the accuracy of the non-i.i.d.-aware case has considerably improved compared to the non-i.i.d.-blind scenario that does not enforce the FoV constraint derived in (22). The difference, in some cases, is above 10% which is very significant in clustering and demonstrates the critical importance of properly regulating angular separation between REs through mathematical formulation of the correlation threshold.

Comparing the last 2 figures, we can see that in contrast to Figure 12, the improvement in accuracy in Figure 11 is not significant. However, it is important to note that for many applications, even a slight improvement in accuracy is critical. For example, in the context of smart manufacturing, this has the potential to reduce miss-clustering in defect detection. Similarly, in the field of healthcare, marginal gains have been shown to enhance diagnostics reliability [47,48].

## 8. Conclusions

This work presented a cooperative framework for partial task offloading in Edge-AI systems, leveraging stochastic geometry to capture spatial randomness and to guide correlation-aware resource allocation. A key methodological contribution was the derivation of a closed-form upper bound on spatial correlation, which enabled constraints ensuring that only relevant and timely contributions are included in the global model. We further formulated and solved a joint optimization problem over task assignments, offloading ratios, and learning parameters, and proposed a practical PGD method. Through feasibility analysis and simulations, we demonstrated that the framework effectively addresses the challenges of latency guarantees, accuracy, and scalability in heterogeneous MEC environments. In particular, the results confirm that explicitly handling non-i.i.d. data distributions and spatial correlations leads to superior performance compared to baseline models where learning and resource allocation are decoupled.

## Figures and Tables

**Table 1:** Comparison of related works with our proposed framework.

Ref.	Method	Main Contribution	Comparison with Our Work
[27]	Matching-based allocation in fog IoT	Stable parallel sub-task execution, latency reduction	Focuses on fog parallelism, our work integrates spatial correlation and non-i.i.d. modeling in MEC
[20]	Multi-queue scheduling with Actor–Critic DRL	Dependent task completion, energy efficiency	Addresses task dependencies, our work emphasizes correlation-aware offloading and robustness to heterogeneous data
[22]	Lyapunov-based stochastic optimization	Joint offloading and resource allocation with latency/queue guarantees	Provides stability analysis, our framework couples delay guarantees with PGD-based optimization
[28]	Decentralized Sequential Neural Network (DDSNN)	Lightweight inference across low-power devices	Focuses on TinyML inference; our work targets MEC task offloading with stochastic geometry and learning integration
[21]	Adaptive local updates in heterogeneous FL	Handles device heterogeneity by adjusting update counts	Considers computational diversity, our approach also incorporates delay constraints and correlation-aware offloading
[18]	Client selection strategy for FL	Faster convergence, reduced communication overhead	Optimizes participant choice, our framework integrates delay guarantees and data heterogeneity in MEC
[29]	Adversarial FL with Earth Mover’s Distance	Improves global adaptation under non-i.i.d. data	Focuses on privacy-preserving FL, our work extends EMD to MEC with offloading and latency constraints
[30]	Label-invariant knowledge distillation in FL	Mitigates label skew via teacher-student framework	Addresses label heterogeneity, our framework also accounts for spatial correlation, partial offloading, and delay guarantees
**Our Work**	PGD-based joint optimization in MEC	Cooperative partial offloading, EMD-based robustness to non-i.i.d., stochastic geometry analysis, reduced CS load	Provides unified framework coupling task offloading, correlation modeling, and delay-aware learning optimization

**Table 2:** List of system model parameters.

Symbol	Description
RE	Set of requesting entities (task generators)
SE	Set of serving entities (edge servers)
$\lambda_{\mathrm{RE}}$	Spatial density of REs (devices/m^2^)
$\lambda_{\mathrm{SE}}$	Spatial density of SEs (devices/m^2^)
|A|	Total coverage area
$D_{r}$	Task size of RE *r* (bytes)
$C_{s}$	Computing capacity of SE *s* (CPU cycles/s)
$\hat{C}$	Computing capacity of central server (CS)
*L*	Maximum sensing/coverage range of REs (m)
$\phi_{r}^{t}$	Viewing angle of RE *r* at time slot *t*
$\tilde{\varphi }$	Angular width of the field of view (FoV)
φ	Angular displacement between time slots
κ	Correlation threshold among REs’ FoVs
f(k)	PPP probability of observing *k* nodes in area |A|

## Data Availability

The source code and data of this paper can be found in [43].

## Appendix A

We assume that the class distribution for each RE *r* at time *t* satisfies the normalization condition:

(A1) $$\begin{array}{c} \sum_{k}{\hat{P}}_{r,k}^{\left(t\right)}=\sum_{k}P_{r,k}^{\left(t\right)}=1,\;\forall t,\forall r. \end{array}$$

Assume that at time slot t−1, only $K^{\prime }<K$ classes have been observed (i.e., $P_{r,k}^{\left(t-1\right)}>0$), while the remaining $K-K^{\prime }$ classes are unseen. To model the evolution of the class distribution over time while maintaining the normalization constraint, we define the estimated distribution at time *t* as follows:

(A2) $$\begin{array}{c} \sum_{k}{\hat{P}}_{r,k}^{\left(t\right)}=e^{-\kappa_{\mathrm{time}}}\underset{=1}{\underset{︸}{\left(\sum_{k=1}^{K^{\prime }}P_{r,k}^{\left(t-1\right)}\right)}}+\left(K-K^{\prime }\right)\epsilon =1,\;\forall t,\forall r, \end{array}$$

where $\kappa_{\mathrm{time}}$ is a decay parameter controlling the memory of previous distributions, and ϵ is obtained by $\epsilon =\frac{1-e^{-\kappa_{\mathrm{time}}}}{K-K^{\prime }}.$

## Appendix B

To justify the validity and dimensional consistency of (22), this appendix provides the derivation of the spatial–directional correlation function used in the paper. We consider a homogeneous field of REs distributed according to a Poisson process within a circular region of radius *L*. Each RE observes its environment within a FoV characterized by an angle $\phi_{r}$ and a total width $\tilde{\varphi }$. Let two REs, located at $l_{r}$ and $l_{r^{\prime }}$, have an angular separation $\Delta \phi =|\phi_{r}-\phi_{r^{\prime }}|$ and Euclidean distance $d_{r,r^{\prime }}=∥l_{r}-l_{r^{\prime }}∥$.

Following isotropic Gaussian field models in spatial statistics [49,50] and stochastic geometry [51], the pairwise correlation between these REs can be expressed as

(A3) $$\begin{array}{c} C\left(d_{r,r^{\prime }},\Delta \phi \right)=\exp\;\left(-\frac{d_{r,r^{\prime }}^{2}}{2\ell_{s}^{2}}\right)\exp\;\left(-\frac{1-\cos\Delta \phi }{2\sigma_{\phi }^{2}}\right), \end{array}$$

where $\ell_{s}$ denotes the spatial correlation length and $\sigma_{\phi }$ the angular correlation parameter, both ensuring dimensional consistency.

For uniformly distributed REs, the average correlation over spatial and angular randomness can be written as

(A4) $$\begin{array}{c} \bar{C}=\frac{1}{L^{2}\tilde{\varphi }}\int_{0}^{\tilde{\varphi }}\int_{0}^{L}\int_{0}^{L}C\left(d_{r,r^{\prime }},\Delta \phi \right)\;dl_{1}\;dl_{2}\;d\Delta \phi . \end{array}$$

While the above integral does not admit a closed-form solution, a tractable and physically meaningful approximation can be obtained by replacing $d_{r,r^{\prime }}^{2}$ with its expected value under the uniform distribution, $E\left[d_{r,r^{\prime }}^{2}\right]=L^{2}/3$. This substitution gives

(A5) $$\begin{array}{c} C\left(\Delta \phi \right)\approx \kappa_{s}\cdot \exp\;\left(-\frac{1-\cos\Delta \phi }{2\sigma_{\phi }^{2}}\right),\;\kappa_{s}=\exp\;\left(-\frac{L^{2}}{6\ell_{s}^{2}}\right), \end{array}$$

which depends only on the normalized spatial scale $L/\ell_{s}$ and the angular separation Δϕ. This simplification yields a closed-form, dimensionless expression that is well suited for system-level design.

To ensure that the correlation between any two REs remains below a desired threshold κ∈(0,1], we impose C(Δϕ)≤κ. Using (A5) and solving for Δϕ yields

(A6) $$\begin{array}{c} \Delta \phi^{*}=\arccos\;\left(1-2\sigma_{\phi }^{2}\ln\left(\frac{\kappa_{s}}{\kappa }\right)\right). \end{array}$$

Therefore, to suppress excessive task similarity across users, the FoV width should satisfy

(A7) $$\begin{array}{c} \tilde{\varphi }\leq \Delta \phi^{*}=\arccos\;\left(1-2\sigma_{\phi }^{2}\ln\left(\frac{\exp(-L^{2}/6\ell_{s}^{2})}{\kappa }\right)\right). \end{array}$$

This condition provides a physically interpretable trade-off between sensing range, allowable correlation, and FoV overlap. The approximation has been numerically validated and shown to remain within a small deviation of the full triple integral (A4), confirming its suitability for system-level design and analysis.
